# Understanding Tricky Cellular and Molecular Interactions in Pancreatic Tumor Microenvironment: New Food for Thought

**DOI:** 10.3389/fimmu.2022.876291

**Published:** 2022-05-31

**Authors:** Antonio Agostini, Arturo Orlacchio, Carmine Carbone, Ilaria Guerriero

**Affiliations:** ^1^ Medical Oncology, Department of Medical and Surgical Sciences, Fondazione Policlinico Universitario Agostino Gemelli IRCCS, Rome, Italy; ^2^ Medical Oncology, Department of Translational Medicine, Catholic University of the Sacred Heart, Rome, Italy; ^3^ NYU Grossman School of Medicine, NYU Langone Health, New York, NY, United States; ^4^ Biogem, Biology and Molecular Genetics Institute, Ariano Irpino, Italy

**Keywords:** PDAC, TME, ECM, immune escape, immunotherapy

## Abstract

Pancreatic ductal adenocarcinoma (PDAC) represents 90% of all pancreatic cancer cases and shows a high mortality rate among all solid tumors. PDAC is often associated with poor prognosis, due to the late diagnosis that leads to metastasis development, and limited efficacy of available treatments. The tumor microenvironment (TME) represents a reliable source of novel targets for therapy, and even if many of the biological interactions among stromal, immune, and cancer cells that populate the TME have been studied, much more needs to be clarified. The great limitation in the efficacy of current standard chemoterapy is due to both the dense fibrotic inaccessible TME barrier surrounding cancer cells and the immunological evolution from a tumor-suppressor to an immunosuppressive environment. Nevertheless, combinatorial therapies may prove more effective at overcoming resistance mechanisms and achieving tumor cell killing. To achieve this result, a deeper understanding of the pathological mechanisms driving tumor progression and immune escape is required in order to design rationale-based therapeutic strategies. This review aims to summarize the present knowledge about cellular interactions in the TME, with much attention on immunosuppressive functioning and a specific focus on extracellular matrix (ECM) contribution.

## Introduction

Pancreatic ductal adenocarcinoma (PDAC) is a deadly disease with a 5-year overall survival of 10% (1). This solid tumor is characterized by a dense fibrotic tumor microenvironment (TME) constituted by connective tissue, fibroblasts, blood vessels, and immune cells. Notably, PDAC is fueled by the immunosuppressive TME (2), thus revealing that the relationship between cancer progression and immunological evolution of TME is a key point to improve therapies (3). Although several solid tumors show a good response to immunotherapies, PDAC lacks effective treatments due to the continuous changes in the immune TME, where immunosuppressive cells are recruited, such as tumor-associated macrophages (TAMs), regulatory T cells (Tregs), and bone marrow myeloid-derived suppressor cells (MDSCs) (4), that all together help cancer cells to escape immunosurveillance (5). Since 1997, chemotherapy based on gemcitabine as a single agent had been a standard-of-care first-line treatment for more than two decades, but two important clinical trials had shown that combination regimens could guarantee stronger response and longer median overall survival. In detail, PRODIGE and MPACT analyzed the utility to combine several chemotherapeutic agents to increase the efficacy of metastatic PDAC treatment (6–9). FOLFIRINOX (5-fluorouracil, leucovorin, irinotecan, and oxaliplatin) and gemcitabine/nab-paclitaxel are current first-line treatment for PDAC patients with metastasis, but they have been associated with many side effects (10–12). Currently, few novel effective treatments are available for this cancer, despite the fact that patients diagnosed with other solid tumors can rely on several therapeutic strategies, highlighting the need to strengthen the research in this field. Future perspectives for PDAC treatment are looking at the combination of immunotherapeutic and chemotherapeutic agents, aiming to fight cancer progression by multiple approaches.

Despite numerous clinical trials recruiting PDAC patients to test novel therapeutic strategies, a deep understanding of pathological mechanisms driving carcinogenesis is needed. In this context, it is helpful to consider that a complex interaction among cells in the TME orchestrates PDAC progression and determines the scarce success rate of available therapies, due to the limited accessibility to cancer cells.

Recently, stromal, immune, and cancer cell interactions have received much attention, being involved in PDAC progression and immune response modulation. However, it is not completely clear how these cells interact in the TME.

This review aims to summarize lights and shadows of this complicated communication, considering critical mediators that are emerging as important players in pancreatic tumorigenesis and progression. Moreover, a specific focus on the recent therapeutic strategies is also provided, attesting that different combination treatments are entering clinic trials and seem to be promising approaches to improve personalized therapies.

## Molecular Subtypes of PDAC: The Contribution of TME Cells

PDAC mainly develops from pancreatic intraepithelial neoplasia (PanIN) (13), which is denoted by microscopic precursor lesions undetectable with present diagnostic imaging techniques. A small percentage of PDAC originates from pancreatic cystic lesions, such as intraductal papillary mucinous neoplasms (IPMNs) and mucinous cystic neoplasms, and can have different aggressiveness depending on the specific site in the pancreas (13). Histopathological features of PDAC have been widely described over time (14, 15), but this classification does not correspond to precise indications for treatments.

Molecular subtyping of PDAC could be more informative, and the single gene mutations most commonly considered are *KRAS*, *TP53*, *SMAD4*, and *CDKN2A* with a prevalence of more than 50% in patients (16, 17). The progression from PanIN to PDAC is marked by the accumulation of several molecular events: *KRAS* mutations and telomerase shortening are early events that determine the transition from normal duct to PanIN-1; *CDKN2A* mutations are related to PanIN-2 stage; late events, such as *TP53, SMAD4*, and also *BRCA* mutations, lead from PanIN-3 to PDAC, with the consequent progression to metastatic disease (18, 19).

During the last decade, several studies have been published in which whole-genome sequencing and transcriptional profiling analysis were applied on large cohorts of PDAC samples with the aim of dissecting the molecular landscape of PDAC (20–24). This has been possible thanks to the advances in next-generation sequencing technologies and encouraged by the promising results achieved in other tumor types with therapeutic approaches based on a molecular stratification of the patients (25–27). In 2011, Collisson and colleagues performed a first array-based mRNA expression analysis of resected PDAC by epithelium microdissection with stroma exclusion. They proposed three subtypes, namely, classical, quasi-mesenchymal, and exocrine-like, with the quasi-mesenchymal subtype showing high tumor grade and poor survival (28).

In 2015, Moffitt and colleagues completed the molecular subtyping of PDAC samples and metastasis by hybridization arrays, and a subgroup of them by RNAseq. Transcripts derived from normal pancreas and from TME were excluded, defining two PDAC subtypes called basal-like and classical, and two stromal subtypes described as normal and activated, with the last one being associated with a worse prognosis (29).

In 2016, Bailey and colleagues profiled PDAC samples with a wide variety of cellularity by array-based hybridization, describing four subtypes, namely, squamous, pancreatic progenitor, immunogenic, and aberrantly differentiated endocrine exocrine (ADEX) (30). In comparison to Collisson classification, Bailey et al. added the immunogenic subtype by the profiling of the immune infiltrates in the TME, and this is extremely important to identify an ideal therapeutic strategy, especially for immunotherapeutic options.

Transcriptional profiling has been useful and informative for signature mutations in *KRAS, TP53, CDKN2A*, and *SMAD4* that have been confirmed, but, more importantly, new genes have been found mutated or transcriptionally altered, thus uncovering a considerable genetic heterogeneity among PDAC patients (22, 31). For instance, about 10% of pancreatic cancer cases are familiar and show germline inactivating mutations in genes associated with the DNA repair pathways (e.g., *BRCA1/2, ATM*, and *PALB2*) and a subgroup of these patients also have similar germline mutations in epigenetic regulators (e.g., *TET2, DNMT3A*, and *ASXL1*) (22, 32). This suggests that epigenetic changes are an important factor in predisposing individuals to pancreatic cancer.

Moreover, whole exome and genome sequencing exposed the presence of somatic mutations in epigenetic regulators and chromatin remodeling complexes (e.g., *ARID1A/B, PBRM1, MLL2/3/4, KDM6A*, and *SMARCA2/4*) in a significant percentage of PDAC patients (22, 31).

These results further highlight the deep heterogeneity (both molecular and epigenetic) of PDAC, made also evident by the identification of various PDAC subtypes with different molecular and phenotypic characteristics that reflect on prognosis and response to therapies (28–30, 33).

Classical subtype tumors are more differentiated and tend to respond better to chemotherapy and to have better prognosis. On the other hand, basal-like subtype is characterized by high tumor grade, strong chemoresistance and worse prognosis. From a molecular standpoint, these subtypes are associated with distinct gene signatures and epigenetic profiles. Specifically, the classical subtype is characterized by an epithelial differentiation gene signature, while the basal-like subtype shows a more mesenchymal expression profile (21–24, 28–30, 33, 34).

Moreover, the two subtypes show differences in the activity of specific superenhancers (SEs) and their upstream regulators (21). SEs are large clusters of transcriptional enhancers that drive gene expression to control cell identity (35, 36). The main transcription factors (TFs) involved in the regulation of subtype-specific SEs and transcription programs are *MET, MYC*, and the ΔN isoform of the transcription factor *TP63* (ΔNp63) for the basal-like subtype, and *GATA6, PDX1*, and *HNFs* for the classical one (37–41). There is evidence that the activity of these transcription factors is controlled by epigenetic regulators that can not only alter their expression, but also function as transcriptional co-regulators (41, 42).

Somerville et al. demonstrated that the ΔN isoform of the transcription factor *TP63* (ΔNp63) is a master regulator critical for establishing basal-like cell identity in PDAC through enhancer reprogramming, thus promoting tumor growth and metastatic potential (43). Mechanistically, ΔNp63 increases H3K27ac levels at the enhancers of basal-like lineage genes, thus leading to increased transcriptions of genes such as *KRT5/6*, *TRIM29*, and *PTHLH*, which promotes more aggressive PDAC phenotypes (43).

Enhancer reprogramming has also been described as the mechanism underlying *FOXA1*-driven tumor-to-metastasis transition (44). Roe et al. established 3D organoid culture from primary tumors derived from the KPC PDAC mouse model and used ChIP-seq analysis to assess H3K27ac occupancy. Their analysis, complemented with *in vitro* and *in vivo* overexpression experiments, revealed that *FOXA1* is responsible for increasing H3K27ac at specific enhancer regions, thus activating foregut developmental genes that promote anchorage-independent cell growth and invasion. Moreover, *FOXA1* gene transcription is enhanced in the presence of missense mutations of *p53* (p53R172H, p53R245W, and p53R270H) (45). Specifically, KRAS effectors phosphorylates cyclic AMP responsive element binding protein 1 (CREB1) and enable binding and hyperactivation by mutant p53. Consequently, FOXA1 is upregulated and, by promoting β-catenin stabilization, activates the canonical WNT pathway supporting proliferation and metastasis (45, 46).

Pancreatic cancer cells can also remodel the epigenetic landscape by repressing epigenetic modulators in order to upregulate TFs that drive squamous PDAC transcriptional programs. For instance, mutations in the histone H3K27me2/3-specific lysine demethylase 6A (*KDM6A*) are frequently found in the basal-like subtypes (47). Andricovich et al. found that loss of KDM6A in PDAC can directly induce the basal-like subtype by rewiring enhancer chromatin and activating SE regulating ΔNp63, MYC, and RUNX3 (47). Mechanistically, such rewiring is the consequence of the activity of histone type 2 lysine methyltransferases (KMT2), which, as a consequence of KDM6A loss, occupy and activate enhancers of genes supporting the basal-like subtype. KMT2 enzyme families are histone H3K4-specific methyltransferases that mark active gene enhancers with H3K4me1 (48, 49) and indeed increased H3K4me1, and KMT2D occupancies at basal-like supporting elements have been reported in the absence of KDM6A (47).

Taken together, these studies highlight the ability of pancreatic cancer cells to reprogram their epigenetic landscape and subsequent transcription programs to sustain their growth, differentiation, and metastasis.

In addition, recently, several studies have employed single-cell RNA sequencing (scRNA-seq) aiming at further elucidating the complexity of TME in PDAC (**Table 1**).

**Table 1:** scRNA-seq analyses to dissect the molecular complexity of TME in PDAC: a historical summary.

Year	Molecular analysis Samples	Resulting evidence	Reference
2018	scRNA-seq IPMN patients	Low-grade IPMNs are enriched for CTLs and CD4^+^ effector T cells compared to high-grade IPMNs	Bernard et al. (50)
2019	scRNA-seq PDAC patients	Three patient clusters identified: cluster 3 vs. clusters 1 and 2 showed high expression of proliferation markers and worse survival; enrichment of cell cycle, DNA replication, and DNA repair pathways and depletion in several immune/T-cell activation gene sets	Peng et al. (51)
2019	scRNA-seq PDAC patients, murine models	Two immune clusters identified: 1. Myeloid cluster, composed of resident macrophages, M2 macrophages, classic monocytes, cDC1, and two types of Langerhans-like dendritic cells 2. Lymphoid cluster, composed of CD8^+^ T cells, CD4^+^ T cells, Tregs, proliferating Tregs, and NK cells	Elyada et al. (52)
2019	scRNA-seq Murine models of PDAC (KIC, KPC, KPfC)	Two immune clusters identified: 1. Expression of several genes associated with chemokines and inflammation 2. Enriched in MHC-II-associated genes	Hosein et al. (53)
2020	scRNA-seq Human primary tumors and metastatic lesions	Two tumor-infiltrating lymphocyte clusters identified, with no difference between primary tumors and metastases: 1. High levels of markers associated with activation and exhaustion 2. Antigen-inexperienced T cell Two macrophage clusters identified: 1. M2 polarization, expression of genes associated with extracellular matrix production and wound healing processes 2. Expression of genes associated with antigen presentation.	Lin et al. (54)

In a study from 2019, Elyada et al., using both PDAC patient samples and murine models, identified two main immune cell clusters: myeloid and lymphoid (52). Subsequent subclustering showed the presence of six distinct subpopulations within the first group, and five within the second. Specifically, for the myeloid cluster, resident macrophages, alternatively activated M2-like macrophages, classic monocytes, conventional type 1 dendritic cells (cDC1), and two types of Langerhans-like dendritic cells were identified. For the lymphoid cluster, the identified cell types were CD8^+^ T cells, CD4^+^ T cells, Tregs, proliferating Tregs, and natural killer (NK) cells.

ScRNA-seq analysis of the immune cells in the TME has also been employed to show differences between low-grade and high-grade tumors, as well as between primary versus metastatic lesions. In 2018, Bernard et al. performed a single-cell transcriptomic profiling of cystic precursor lesions of PDAC demonstrating that low-grade IPMNs are enriched for CTLs and CD4^+^ effector T cells compared to high-grade IPMNs. At the same PDACs, when compared to IPNMs, show an increased presence of granulocytic MDSCs. This suggests a progressive shift of the microenvironment in a tumor-promoting direction (50). This modulation of the TME by the malignant cells seems to be supported by other studies as well. For instance, Peng et al. identified three PDAC patient clusters, with cluster 3 being characterized by proliferation markers and associated with worse survival compared with patients in the other two clusters. Moreover, differential gene expression analysis showed an enrichment of cell cycle, DNA replication, and DNA repair pathways and depletion in several immune/T-cell activation gene sets in cluster 3 in comparison to clusters 1 and 2 (51). They reported an inverse correlation between high expression of proliferative ductal markers and low expression of T-cell activation markers. This result was then confirmed by immunohistochemistry (IHC), which demonstrated that areas characterized by ductal cells expressing low levels of Ki67 were also characterized by high T-cell infiltration and *vice versa*, thus linking altered ductal cell proliferation and local immune response and suggesting that a combination of cell-cycle inhibitors and immunotherapy could be a valid therapeutic approach (55).

Two novel subtypes of macrophages were identified by Hossein et al. in advanced tumors by applying scRNA-seq to three mouse models of PDAC: Kras^LSL-G12D/+^ Ink4a^fl/fl^ Ptf1a^Cre/+^ (KIC), Kras^LSL-G12D/+^ Trp53^LSL-R172H/+^ Ptf1a^Cre/+^ (KPC), and Kras^LSL-G12D/+^ Trp53^fl/fl^ Pdx1^Cre/+^ (KPfC) (53). Specifically, one subtype expressed several genes associated with chemokines and inflammation, while the other was enriched in major histocompatibility complex II (MHC II)–associated genes.

In regard to the differences in TME immune cells between primary tumors and metastatic lesions, using scRNA-seq, Lin and colleagues compared immune cell population from primary tumor resections with the ones obtained from metastatic biopsies (54). Two tumor-infiltrating lymphocyte (TIL) clusters were identified, showing no difference between primary tumors and metastases. One cluster was characterized by high levels of markers associated with activation and exhaustion, while the second one was representative of naive, antigen-inexperienced T cells. On the other hand, macrophages from primary tumors and metastases clustered separately. While the first displayed a gene signature typical of M2-like polarization (higher levels of genes associated with extracellular matrix production and wound healing processes), the second expressed genes associated with antigen presentation. However, it is worth mentioning that the analyzed metastases were mostly hepatic; therefore, the observed differences may be partially due to the distinct characteristic of liver-resident and pancreas-resident macrophages.

A downside of scRNA-seq is the loss of tissue architecture, which constitutes an obstacle to the study of intercellular interaction. For this reason, complementary approaches like multiplexed immunolabeling or RNA *in situ* hybridization (RNA-ISH) have been developed. Despite having significantly less molecular resolution, they provide spatial information at the single-cell level. One of the earlier attempts was reported by Carstens et al. who were able to simultaneously assess eight markers [Dapi, alpha-smooth muscle actin (α-SMA), collagen I, cytokeratin 8, Foxp3, CD3, CD4, and CD8] on tissue microarrays composed of tissue obtained upon pancreatectomy of 132 patients with PDAC without neoadjuvant therapy (56). Interestingly, they report an independent association between improved patient survival and high infiltration levels of total T cell, CD8^+^ cytotoxic T cell, and CD4^+^ effector T cell (56); however, such association became only significant for the seconds when taking into consideration only a 20-µm radius around each cytokeratin 8-positive cancer cell. Moreover, no correlation was found between α-SMA levels and T-cell infiltration, while collagen I deposition positively correlated with T-cell infiltration, suggesting that desmoplastic stroma does not negatively impact lymphocyte infiltration (57, 58).

A similar approach was employed with a focus on myeloid cells and macrophages by Väyrynen and colleagues (59). Using tissue microarrays generated from 305 primary PDAC specimens, the authors focused on four polarization markers to assess the macrophage polarization status (M1: CD86, IRF1; M2: CD163, CD206). They reported that M1-polarized macrophages were located in significantly closer proximity to cancer cells than M2-polarized macrophages and that a higher density of the latter as well as CD15^+^ARG1^+^ immunosuppressive granulocytic cells was associated with poor patient survival. Moreover, the authors reported interesting associations between myeloid cell densities and alterations in PDAC driver genes, thus further supporting the effect of cancer cell on immune cell modulation in the TME (59).

The integration of ISH techniques with scRNA-seq data has allowed mapping rare cellular subpopulations on a spatial context (60, 61). However, a throughput limitation persists in ISH techniques, avoiding considering it the best spatial approach. Aiming to overcome this limit, recent spatial transcriptomics (ST) methods have been developed, in order to map any transcripts in whole tissue sections using ISH of spatially barcoded oligonuclotides (62). Very recently, Moncada et al. (2020) showed the potential of this breaktrough technology to study PDAC TME composition (63). They used an array-based ST novel approach to deconvolute scRNA-seq on whole tissue by dividing the PDAC samples into two portions: one to be used to obtain a single-cell suspension processed for scRNA-seq; on the second portion, ST was performed to map the expressed transcripts across the tissue. By the integration of the two resulting analyses output, from primary PDAC tumors from different patients, they were able to identify several specific cell types and subpopulations, such as M1 and M2 macrophages, enriched across spatially restricted areas of the tissue.

### Keynote

Understanding molecular features of PDAC can reveal a source of novel targets to be exploited for important advances in PDAC therapy. The improvement of methods and the integration of different technologies can give a comprehensive overview of molecular landscape, during cancer progression and resistance to treatments. The knowledge of genetics, epigenetics, and transcriptomics behind PDAC is the key to targeting crucial pathological mechanisms.

## Immunosuppressive Cells in the TME of PDAC

TAMs derive from the recruitment of CCR2^+^ monocytes to the TME and usually represent the most abundant immune population (64–66). In general, macrophages have distinct states of polarization, which are commonly defined as M1 and M2. The M1 phenotype is associated with pro-inflammatory function and is activated through the classic pathway, by IFNγ or bacterial component stimulation; the M2 phenotype is related to anti-inflammatory function, and is activated by alternative pathways that lead to the suppression of Th1 immune response in favor of the Th2 one (67, 68). As part of the innate immune response, monocytes are recruited in the first phase of cancer onset and differentiate in macrophages able to phagocyte cancer cells, but their function is impaired by several mechanisms of immune escape. TAMs in TME show very heterogeneous features; however, most of them display an M2 polarization state, supporting angiogenesis and tumor growth (69, 70). Cancer cells can adopt mechanisms to evade immune surveillance; an example is to express high levels of the transmembrane protein CD47, which represents the classic signal “don’t eat me”, inducing an anti-phagocytic response (71). Several cancers exploit this immune evasion strategy, and some authors have considered the blockade of CD47 on cancer cells or signal regulatory protein α (SIRPα) on macrophages as a valid therapeutic option (72, 73), also for PDAC to target pancreatic cancer stem cells and as adjuvant immunotherapy for liver micrometastasis (74, 75). TAMs can also secrete in the TME a number of immunosuppressive cytokines, such as IL-6, TGF-β, and IL-10 that are able to suppress CD8^+^ T-cell function (76). Specifically, IL-6 is expressed at high levels in PDAC, and its increasing circulating level is associated with advanced disease and poor prognosis (77). The inhibition of IL-6 signaling along with CD40 blockade is able to revert the TME to support an antitumor immune response, by reducing TGF-β activation and fibrosis deposition due to a decreased collagen type I production (78). Moreover, chemokines such as CCL2, CCL17, CCL20, and CCL22 induce the recruitment of Tregs to the tumor sites, activating their regulatory function by IL10 and TGFβ signaling, leading to the accumulation of Tregs and impairing the migration and activation of T cytotoxic effector cells (79–82). TAMs can also express arginase I that is involved in reducing L-arginine in the TME impairing T-cell function (83). TAMs are also responsible for a reduced NK cell function, due to the secretion of the above-mentioned cytokines resulting in a limited production of IFN-γ, perforin, and IL-12 by NK cells, which determines a lower cytotoxicity and proliferation in the TME (84). TAMs are able to reduce NK cell functioning also by direct cell–cell interactions, since PDL-1 expressed on TAMs can bind to PD-1 on NK cells, avoiding the activation of their cytotoxic receptors (85).

Similarly to TAMs, neutrophils can also show heterogeneity in the TME, showing a different state of activation and consequent function. Neutrophils take part in early inflammatory response, being able to produce and secrete many cytotoxic compounds and also reactive oxygen species (ROS), in order to kill stromal cells in the TME (86). By secreting a high number of chemokines, such as CCL2, CCL3, CCL19, and CCL20, neutrophils can drive the immune response, recruiting monocytes and DCs, NK cells, and T-helper type 1 (Th1) and type 17 (Th17) cells to the inflamed tissues (87, 88). Despite a clear pro-inflammatory function, neutrophils can change to a pro-tumor profile. Thus, the population of tumor-associated neutrophils (TANs) can be considered dichotomous, showing an N1 or N2 profile, comparably to TAMs. The N2 profile sustains tumor growth by the activation of TGF-β signaling (89). Moreover, pancreatic cancer cells can recruit TANs by secreting chemokines of the CXC family, specifically CXCL6 and CXCL8 or CXCL1–3 and CXCL5–8 that are recognized by CXCR1 or CXCR2 receptors expressed on neutrophils (90). High levels of CXCR2 have been associated with tumor size in PDAC (91), and a high number of TAN infiltrates can be considered an indication of higher malignancy and worse prognosis in PDAC, considering the expression of the CD177 neutrophil marker (92). A very recent study has demonstrated that lorlatinib, an FDA-approved ATP-competitive small-molecule tyrosine kinase inhibitor, is able to inhibit the growth of PDAC at primary and metastatic sites, through the regulation of neutrophil development and recruitment and by constraining neutrophil-induced tumor growth in the TME, in preclinical murine models of PDAC (93).

Myeloid-derived suppressor cells (MDSCs) are immature myeloid cells with heterogeneous features; in fact, they can be phenotypically similar to monocytes defining the subpopulation of mononuclear or monocytic (M-MDSCs or Mo-MDSCs), or they can be more like neutrophils and are called polymorphonuclear (PMN-MDSCs) or granulocytic (G-MDSCs or Gr-MDSCs) (94, 95). MDSCs can exploit their strongly immunosuppressive functions by several mechanisms. One of them is to reduce T-cell proliferation through the increased PD-L1 expression that binds to the PD-1 receptor on T cells inhibiting their activation and self-tolerance (96, 97). Moreover, MDSCs may positively regulate the expansion of immunosuppressive Tregs by IL-10-induced TGF-β and IFNγ production (98) or by the secretion of reactive oxygen species, such as ROS, Arg1, and iNOS (99). MDSCs can proliferate and accumulate in the TME through the stimulation received by some cytokines and chemokines produced after Yap signaling activation (100). High levels of MDSCs, both in peripheral blood and as tumor infiltrates, have been associated with low overall survival and metastasis development in patients, even if their immunosuppressive function is not common for all PDAC patients. Specifically, a detailed gene signature has revealed that immunosuppressive MDSCs can be defined as circulating STAT3/arginase1-expressing CD14^+^ cells (101). MDSCs can also directly promote tumor growth and angiogenesis by MMP9 and VEGF secretion; in fact, they can produce high levels of matrix metalloproteinases that are able to dissolve extracellular matrix (ECM) and allow cancer cells to migrate and invade other tissues (102). In addition, through the secretion of high levels of VEGF and basic fibroblast growth factor (bFGF), they can sustain angiogenesis (103).

Tregs represent an immune subset population of T lymphocytes CD4^+^CD25^+^FOXP3^+^ with an immunosuppressive function that is present in the TME of both PanIN and PDAC (104). A high number of Treg infiltrates in the tumor has been correlated to poor prognosis and metastasis development (105, 106). Tregs can interact with CD11c^+^ DCs determining their reduced expression of MHC II, co-stimulatory molecules (CD40 and CD86), and indoleamine 2,3-dioxygenase (IDO), suppressing IFN-γ production and finally T-cell activation (107). Moreover, PDAC patients have shown an imbalance in the number of Tregs and Th17 cells, with a notable increase in their ratio, determining important changes in cytokine production, such as higher levels of IL-10 and TGFβ, and lower levels of IL-23, INF-γ, and IL-17, with a consequent T-cell inactivation (108).

On the other side, very recently, Tregs have been correlated to an antitumorigenic effect by immune response stimulation during pancreatic carcinogenesis. The depletion of Tregs in mouse models of spontaneous tumorigenesis both before and after the onset of PanIN determined a strong tumor progression, and in murine models of invasive tumors, the depletion of Tregs was not able to control cancer growth. A compensatory mechanism to increase other CD4^+^ T cells and also immunosuppressive myeloid cells has been demonstrated as a consequence of Treg depletion (109, 110).

**Figure 1:**
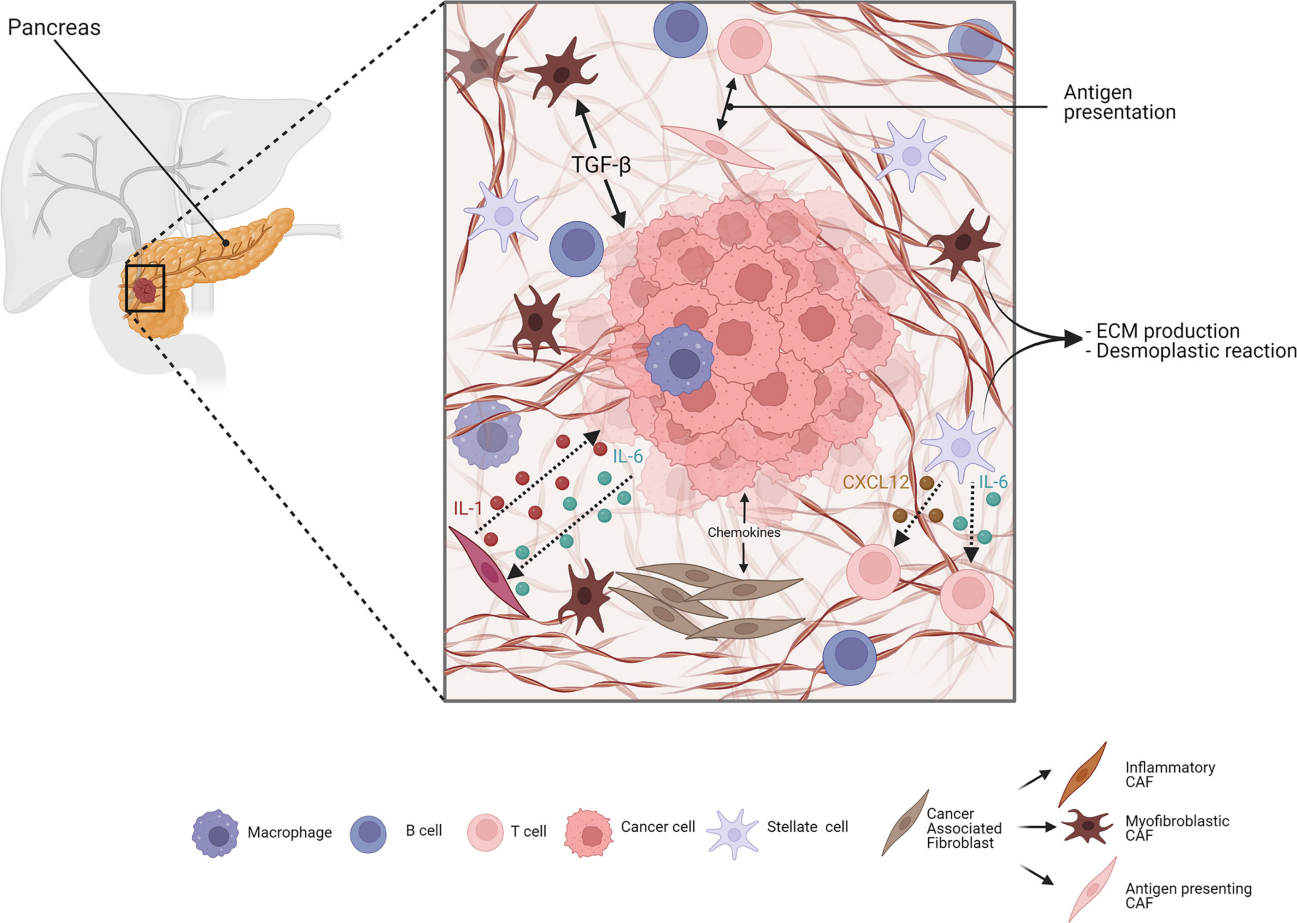
Simplified scheme of stroma-mediated interaction in PDAC. Cancer-associated fibroblasts (CAFs) are crucial elements of the pancreatic ductal adenocarcinoma (PDAC) stroma. They include different subtypes: myofibroblastic, inflammatory, and antigen-presenting subtypes. Both cell–cell and paracrine interaction CAFs and PDAC cells are involved in manipulating the stroma. The cancer cells can induce, through transforming growth factor-β (TGFβ) signaling, the surrounding CAFs to adopt a myfibroblastic phenotype. Similarly, cancer cells produce IL-1, reprogramming CAFs to inflammatory CAFs, which, in turn, produce chemokines like IL-6 and sustain cancer growth. Antigen-presenting CAFs, expressing MHC class II molecules, modulate the immune cells in the stroma. Pancreatic stellate cells (PSCs) are mainly responsible for ECM deposition during PDAC, and are also able to modulate the immune response through the production and secretion of cytokines, such as CXCL12 and IL-6, negatively affecting T-cell activity and migration. Adapted from “PDAC histology” by BioRender.com (2022). Retrieved from https://app.biorender.com/biorender-templates.

Besides immune cells, stromal cells in the TME can also influence cancer progression and immune response. A scheme of stroma-mediated interaction in PDAC is proposed in **Figure 1**. Cancer-associated fibroblasts (CAFs) play a crucial role in this context, since they are responsible for ECM deposition and remodeling. They are the most abundant population representing up to 85% of all stromal cells, and are also involved in a complex crosstalk with cancer and immune cells (111). Fibroblasts do not show a characteristic expression of specific surface markers; thus, it is very difficult to give a precise definition of their origin and whether they convert in CAFs during tumor progression. In a human cancer biopsy, CAFs can be identified for exclusion due to the absence of epithelial, endothelial, and leukocyte markers; the lack of molecular mutations by cancer cells; and the characteristic elongated morphology (112). A common consensus is that CAFs have a tumor-suppressive function at the early state of tumorigenesis, since depletion of an αSMA^+^ subset of fibroblasts in a PDAC mouse model led to undifferentiated tumors with enhanced hypoxia, increased tumor invasion, and decreased animal survival (113), but during tumor progression, they can dynamically change their role. CAFs are able to produce fibrotic compounds, such as collagens, hyaluronic acid, and fibronectin, contributing to ECM deposition (114) (better discussed below). In addition, CAFs can secrete chemokines, cytokines, growth factors, miRNAs, extracellular vesicles, and metabolites to communicate with cancer cells and other TME players to promote tumor progression (115). Over time, different CAF subpopulations have been defined: myofibroblastic CAFs (myCAFs) that express α-SMA and are responsible of TGFβ production; inflammatory CAFs (iCAFs) that produce inflammatory mediators, such as cytokines, chemokines, and complement complex; and antigen-presenting CAFs (apCAFs) that express CD74 and MHC class II and interact with CD4^+^ T cells. This apCAF subpopulation, however, lacks the necessary co-stimulatory molecules to activate T cells, and is, therefore, supposed to have an immunosuppressive role by acting as a decoy tilting the ratio of CD8^+^ to Tregs.

Many authors have confirmed this classification by scRNA-seq, in both mouse and human tissues (50, 52, 53, 116, 117). iCAFs are associated with an activity of immune modulation, which is crucial during PDAC progression, and are activated in a paracrine manner by cancer cells through the secretion of stimulating factors, but they are located distant from cancer cells and myCAFs. Once activated, iCAFs can produce inflammatory mediators, such as IL-6, IL-8, IL-11, CXCL1, CXCL2, CXCL12, and leukemia inhibitory factor (LIF). Moreover, they show an activation of several inflammatory pathways, such as IFN-γ response, TNF/NF-κB, IL-2/STAT5, and IL-6/JAK/STAT3 signaling in humans (118). iCAFs can modulate the immune response at different levels, inducing M2 polarization of TAMs, accumulating MDSCs, TANs, regulatory B-cells (Bregs), and Th17 cells in the tumors, but also directly decreasing CD8^+^ T cells through the production of the βig-h3 stromal protein in a TGF-β-dependent manner (119). Recently, an additional subtype has been identified in loose-type stromal PDAC compared to dense-type stromal PDAC and named meCAFs, representing a highly activated metabolic state and associated with a poor prognosis but a better response to immunotherapeutic inhibition of PD-1 (120). In addition, a new CAFs subset, named complement-secreting CAFs (cs-CAFs), has been identified in early PDAC by scRNA-seq, showing high enrichment for the components of the complement system, such as C3, C7, CFB, CFD, CFH, and CFI, and being able to modulate the immune response in the tumor (121).

Interestingly, these CAF subpopulations show a level of plasticity being able to shift among the different phenotypes (52), thus suggesting that TME modulation to improve therapies based on immunological agents is theoretically possible.

Pancreatic stellate cells (PSCs) are resident cells in the pancreas and are mainly responsible for fibrosis deposition during PDAC (122), and their crucial role in pancreatic cancer progression has been investigated more deeply in recent years. PSCs were isolated for the first time in 1982 by Watari and colleagues who identified, in murine pancreas, cells containing vitamin A droplets after an excess of retinoid administration (123). In addition to several physiological functions, such as pancreatic architecture maintenance, tissue homeostasis, induction of amylase secretion, phagocytosis, and ECM turnover (124), their contribution to pathological mechanisms has also been elucidated, leading to the confirmation that PSCs can influence the dense desmoplastic reaction, tumor progression, metastasis, and resistance to therapies (125). They are in a quiescent state and can respond to different stimuli, such as cytokines/transcription factors, non-coding RNAs, oxidative stress-related factors, hyperglycemia, and ion channels and calcium signaling, to perform their activities (126). Activated PSCs (aPSCs) acquire a myofibroblast-like phenotype and produce ECM. In addition to many physiological functions, aPSCs can play important roles also in pathological conditions, such as PDAC, being responsible for the abundant desmoplastic reaction that surrounds cancer cells reducing accessibility to drugs. During early tumorigenesis, an intense communication between stromal and cancer cells induces the reprogramming of mesenchymal cells, and aPSCs can represent a valid cellular source of CAFs (127). Despite these two populations expressing similar markers, nowadays they are considered as separate cellular entities; in fact, in experiments of three-dimensional co-culture systems that reproduce the interactions between CAFs and cancer cells, two spatially separated, mutually exclusive, dynamic, and phenotypically distinct CAF subtypes have been identified, but the difference between aPSCs and CAFs still represents an important topic of discussion (116). Besides the fibrotic activity, aPSCs are also able to regulate the immune response during PDAC progression through the production and secretion of cytokines, such as CXCL12, impairing the migration of CD8^+^ and CD4^+^ T cells, NK cells, and Tregs to the juxtatumoral compartment in proximity of the tumor site (57). By several mechanisms, aPSCs can suppress T-cell activity through IL-6 secretion, i.e., inhibiting T effector cell migration, activating Tregs and TAMs, and impairing the balance in the Tregs/T effectors ratio (128).

During the last few years, many authors have contributed to describe the cellular heterogeneity in PDAC, but much is to be learned about how stromal cells, such as CAFs, are able to modulate cancer cells. Recently, Ligorio et al. identified a single-cell population that can switch towards invasive and proliferative phenotypes, marked by MAPK and STAT3 activation (129). This elegant work combined scRNA-seq and proteomics to highlight that CAFs play an important role in modulating cancer cell heterogeneity, and findings obtained in model systems were then translated to primary human tumors, in order to contextualize these cellular populations in the architecture of PDAC tissue. Around 2015, some authors had demonstrated that PDAC is the result of a mosaic in which cancer cells are “tumor islands” and CAFs represent the “sea” all around them. This nice view led to the convincement that the interaction between PDAC cells and CAFs are not strictly defined as stimulatory or inhibitory, but modulations of stromal content can determine a different behavior in specific tumor areas (130–132). Very recently, Grünwald and colleagues defined subTMEs as functional units with specific epithelial and immune phenotypes that are able to influence the progression of PDAC (133). SubTMEs can be classified into “deserted” regions (regions characterized by the presence of spindle-shaped fibroblasts), “reactive” regions (regions with plump fibroblasts containing enlarged nuclei), and “intermediate” regions (with mixed features and an intermediate level of both characteristics). Molecular and immune features are different in the three types of subTMEs, but the key message of this study is the involvement of stroma in influencing the response to chemotherapy as well. The authors showed that the deserted subTME has a chemoprotective role, associated with a poor response, leading to the conclusion that future approaches aimed to attenuate the deserted TME state could be able to improve therapy outcome.

### Keynote

Intratumoral heterogeneity is the major obstacle for effective PDAC therapies. Tricky cellular interactions support tumor progression and resistance to current treatments. The intuition of different types of communication of cancer cells with stromal and immune compartments, in several spatial architecture contexts, is the starting point to understand that PDAC needs a novel approach. Taking into consideration the multiple faces of the disease, opposing pro-tumor behaviors and enhancing tumor-suppressive ones, could be a valuable strategy to fight PDAC.

## The Emerging Role of ECM Components in Immune Escaping

A desmoplastic reaction is the deposition of a dense layer of fibrotic ECM that happens as a response to an insult of different nature such as tissue damage or neoplastic growth. Desmoplasia is in fact a hallmark of PDAC, where it probably originates as an attempt to restrain neoplastic cells (134). In fact, several studies showed that impairing the stroma deposition lead to a more aggressive disease (113, 135, 136). PDAC cells, however, remodel ECM to escape the confinement and interact with many ECM proteins to support its growth. It is well known that the protein-rich and collagen-based ECM plays an important role in PDAC oncogenesis (137). This dense matrix is composed of type I, type III, and type IV collagens, glycoproteins, proteoglycans, and glycoaminoglycans that altogether support tumor progression, metastatization, and therapy resistance (138, 139).

Recently, several studies also showed that ECM components also play a role in immune regulation.

In PDAC, collagens are the most abundant ECM proteins where they form the main scaffold for the TME. The binding of ECM collagens with integrins and receptors such as DDR-1 expressed on the surface of neoplastic cell promotes proliferation and migration of PDAC (137). Collagen overproduction and consequent fibrosis seems to be inversely correlated with immune infiltration in PDAC, mainly by providing a barrier for immune cells and activating signaling that promotes immune escape. Focal adhesion kinase (FAK) is the main driver of collagen production in PDAC where it is hyperactivated. The expression of this protein in PDAC correlates with fibrosis and immune suppression (140). Loss of FAK in PDAC caused not only a decrease in collagen deposition but also an increase in effector T-cell infiltration in PDAC models (140). Sharma et al. (141) targeted the hexosamine biosynthesis pathway (HBP), a shunt pathway of glycolysis with 6-diazo-5-oxo-l-norleucine to disrupt collagen deposition in the TME, causing an increase in immune infiltration and an enhancement of immune checkpoint inhibitory (ICI) therapy, such as anti-PD1. Deng et al. (142) showed that the binding of collagen I to DDR1 promoted PDAC growth, and it was also the major stimulus for CXCL5 production mediated by a DDR1/PKCθ/SYK/NF-κB signaling cascade. CXCL5 production and secretion resulted in the recruitment of TANs, which not only favored immune suppression but also supported cancer cell invasion and metastasis by formation of neutrophil extracellular traps. These traps are web-like extracellular fibers formed by neutrophils in the ECM, consisting of chromatin DNA filaments, lactoferrin, myeloperoxidase (MPO), histones, and elastase that are able to activate PDAC invasion and also cause apoptosis of cytotoxic T cells (142).

In addition to collagen, the TME also contains high levels of glycoproteins that confer an immunosuppressive status.

Galectins are small glycoproteins that actively support cancer growth and also immune escaping. These proteins are potent negative regulators of the immune cell functions, and they are highly expressed in cancer where they favor immune escaping mainly by inducing CD8^+^ T-cell death (143). Galectin-1 has been found to be upregulated in the PDAC, and is lowly expressed in long-term (≥10 years) survivors (144). Orozco et al. showed that loss of Galectin-1 in Ela-KrasG12Vp53^−/−^Lgals1^−/−^ murine models leads to a reduced stromal activation and favored a transition in an immune permissive TME causing an effector T-cell infiltration (145). Moreover, Galectin-1 can be secreted by aPSCs mediating the immunosuppression of CD8^+^ T cells and promoting T-cell apoptosis, contributing to the immunosuppressive TME (146). Galectin-3 is also secreted by PDAC in the TME, where it inhibits T-cell proliferation (147). Zhao et al. (148) demonstrated that Galectin-3 released by PDAC stimulates the production of the M2 macrophages inducer IL-8 on PSCs *via* ITGB1/NF-κB signaling. Daley et al. (149) showed that Galectin-9 is also present in PDAC TME where it promotes tumor progression with its ligand Dectin-1. This Galectin ligand is a member of the C-type lectin family of pattern recognition receptors and is present on the surface of myeloid-monocytic lineage cells, especially in macrophages. Dectin-1 is highly expressed in PDAC TAMs, where it promotes the M2 phenotype upon activation by ligation with Galectin-9. Daley et al. showed that anti-Galectin-9 immunotherapy triggered an immune reprogramming in TAMs favoring the M1 phenotype and also provoked an increase in immune infiltration and consequent tumor reduction. This finding paves the way for the development of new treatment strategies for PDAC. In fact, Galectin-9 is also known to be a potent stimulator of T-cell exhaustion and a major cause for immunotherapy failure. Yang et al. (150) showed that Galectin-9 binds both PD-1 and TIM-3 causing both cell apoptosis and T-cell exhaustion in several types of tumors, and they also demonstrated that anti-Galectin-9 immunotherapy was an effective treatment.

Mucins are a family heavily glycosylated proteins that are involved in many physiological mechanisms (151, 152). Mucin secretion is the main characteristic of PDAC precursor lesions (IPMNs) (153). Mucins are also highly expressed in PDAC TME; in fact, most of the recent studies that utilized scRNA-seq to characterize PDAC samples identified a cluster of mucin-producing cells especially in the patients with a more aggressive disease (51, 154, 155). This evidence suggests that mucins play a major role in PDAC carcinogenesis, not only supporting PDAC development by activating several oncogenic pathways, but also sustaining cancer cells to escape the immune surveillance by multiple mechanisms.

MUC1 has been associated with a decreased interaction of the NK cell receptor (NKG2D) with the tumor-associated ligand MICA (major histocompatibility complex class I-related chain A) by the involvement of Galectin-3, which is differentially expressed in pancreatic cancer (156, 157). In detail, Galectin-3 can bind the NKG2D-binding site of MICA through modified core 2 O-glycans of MUC1, thus inactivating NK cells and inhibiting TNF-mediated apoptosis of cancer cells, promoting the development of distant metastasis (158, 159). Moreover, the purification of glycosylated MUC1 from ascitic fluid of pancreatic patients have demonstrated that this mucin can influence DC maturation, due to the limited processing and presentation that retains MUC1 into the early endosomes (160). DCs can also express MUC1 on their surface, impairing Toll-like receptor (TLR) activation (161); in fact, the deletion of *MUC1* gene induces DC response through the activation of TLR4 and TLR5 and the production of co-stimulatory molecules, such as CD40, CD80, and CD86, in addition to the secretion of pro-inflammatory cytokines, such as TNF-α and VEGF (162). Also, MUC2 is able to regulate DC response by decreasing pro-inflammatory cytokines and increasing the secretion of IL-10 and TGFβ1, leading to an increased Treg recruitment (163). On the other side, MUC4 expressed by pancreatic cancer cells induces the apoptosis of cytotoxic T cells in a Fas-independent manner, reducing immune response (164).

Mucins have also been related to metastasis development, due to their deregulated glycosylation that leads to the expression of specific structures on their surface, named T, sTn, sLea, and sLex structures (165). MUC1, MUC2, MUC4, and MUC16 can express these structures functioning as ligands for selectins that are expressed on the surface of leukocytes and platelets, inducing the formation of aggregates and metastasis (166, 167).

MUC5AC determines the suppression of antitumor function of neutrophils, enhancing tumor progression and metastasis. Since IL-8 produced by cancer cells induces neutrophil migration, it has been demonstrated that MUC5AC silencing is able to increase IL-8 production and neutrophil activation in pancreatic cancer cells, showing the important role of this mucin in modulating immune response (168). On the other hand, MUC16 has been associated with long-term survival of pancreatic cancer patients, inducing the activation of T cells reactive to MUC16 neoantigens in response to primary tumors, which are progressively lost during metastasis development (169), attesting that mucin activities are very complex and are strictly related to specific cancer contexts showing different interactions among stromal, cancer, and immune cells.

Furthermore, TME in pancreatic cancer is strongly hypoxic, and PSCs are mainly responsible for pH and oxygen level modulation. In an acidic pH and hypoxic environment, PSCs, in turn, increase the secretion of HGF that can activate MET signaling in PDAC cells. MUC20 can contribute to cancer progression since hypoxia and low pH upregulate MUC20 expression that is able to physically interact with the MET receptor, being a crucial mediator between PSCs and cancer cell communication (170). In addition, hypoxia impairs immune cell function modulating both innate and adaptive immune response, by transcriptional regulation *via* hypoxia-inducible factors (HIFs) (171) and MUC1 is able to stabilize HIF-1α by reducing the intracellular levels of α-ketoglutarate (172).

In the last decade, the perspective about ECM in PDAC changes from an inert material to a key regulator of tumor progression. It is clear now that ECM components’ ratios and quality are finely regulated by PDAC, which uses these molecules to communicate with TME cells and keep immunity at bay. The general view now is that ECM provides a barrier that not only protects PDAC cells physically, but also provides a plethora of immunesuppressive signals. A huge effort has been made to develop new strategies to disrupt these ECM tumor-promoting functions; some of these sound promising, while many failed. Probably, the main reason is that we are still missing many pieces of knowledge about the complex interactions happening in the ECM. New technologies may help us in the near future in this context. The arising technologies of ST and proteomics will give us an unprecedented look into PDAC. The two main spatial technologies Visium (10X Genomics) and GeoMx (Nanostring) are able to map on a histological image the entire transcriptome and the expression of hundreds of proteins simultaneously at a resolution of few dozens of microns, helping researchers to precisely identify and characterize the myriad of interactions that happen in PDAC TME. Moreover, in 2022, two new spatial technologies have been presented, the Xenium (10X Genomics) and CosMx Spatial Molecular Imager (Nanostring) that will move the ST and proteomics at a single-cell and even sub-cellular level resolution, increasing exponentially the understanding and knowledge of PDAC ECM interactome in the years to come.

### Keynote

The hypothesis of a crucial role played by ECM in PDAC progression is a well-demonstrated thesis. Exploiting the ECM, with all the signals supporting tumor growth and helping cancer cells in immune evasion, is a successful approach. However, we need a deeper understanding of specific mechanisms and interactions in the TME. Many studies are focusing on this aspect and future directions are all aimed to compose the puzzle, piece by piece.

## Therapeutic Strategies: How Can We Harness Our Knowledge About TME to Improve PDAC Treatment?

Current treatment options for PDAC are very limited in their efficacy. Chemotherapy with gemcitabine as a single agent has been used for many years (6), but the overall survival of patients remained extremely low; thus, the combination therapy with different chemotherapeutic agents became more effective and entered the clinical practice. Gemcitabine/nab-paclitaxel and FOLFIRINOX (5-fluorouracil, leucovorin, irinotecan, and oxaliplatin) are still valid options, but they are associated with many side effects (10, 12). Recently, a novel second-line treatment based on nanoliposomal Irinotecan (Nal-IRI) proved to be effective. The NAPOLI1 trial (173, 174) showed the efficacy of Nal-IRI in combination with 5-fluorouracil and leucovorin to increase both overall survival and progression-free survival (PFS) in both non-metastatic and metastatic patients. The HOLIPANC trial (175) proved that neoadjuvant combination of Nal-IRI, oxaliplatin, 5-fluouracil, and folinic acid (NAPOX) had a considerable antitumor effect and increased overall survival of patients with a metastatic disease.

Immunotherapy has received much consideration in PDAC, but without reaching high success rate, due to the complex and not fully understood relationship between immune and cancer cells in the TME, as largely described above. Once activated, T cells express PD-1, a transmembrane glycoprotein type I belonging to the immunoglobulin superfamily CD28 that is bound by its ligands, PD-L1 and PD-L2, expressed on antigen-presenting cells (APCs) and cancer cells, resulting in T-cell suppression and exhaustion (176). PD-1 expression is transient and can decrease in the absence of signaling through the T-cell receptor (TCR); otherwise, it is chronically activated in the presence of an epitope target, such as in chronic viral infections and in cancer (177). Less is known about PD-L1 expression on T cells, but recently, it has been demonstrated that its ligation stimulates intracellular signaling with a suppressive activity, similar to PD-1 (178). Moreover, PD-L1 on T cells is able to induce the M2**-**like macrophage differentiation *via* STAT6 signaling and to suppress neighboring effector T cells (178). Cytotoxic T lymphocyte antigen 4 (CTLA-4) is another important inhibitory checkpoint that determines the suppression of the T cells’ response by binding CD80/CD86 on APCs (179). However, the function of CTLA-4 is not completely understood, and some authors have proposed that the CTLA-4 cytoplasmic domain could not be directly involved in the transmission of inhibitory signals, but could be mainly responsible in regulating the access of CD28 receptors to their shared ligands (180). The aberrant overexpression of PD-1, PD-L1, and CTLA-4 is very common in the TME of PDAC, and they still represent good targets for immunotherapy, but targeting one of them as monotherapy approach (immune checkpoint inhibitors ICIs) has not granted good response in PDAC patients as occurred for other types of cancer (181–184). However, recent studies showed that the modulation of the complex cell intrinsic and extrinsic of TME may effectively increase immunotherapy efficacy. Carbone et al. (185) showed that intratumoral injection of the Toll-like receptor 9 agonist IMO-2125 in combination with anti-PD1 activated an immune-suppressive to immune-permissive transition of the TME in PDAC models with both high and low immunogenic potential. Toll-like receptor 9 (TLR-9) is a pattern recognition receptor that is predominantly located in the cytoplasm of DCs, macrophages, NK cells, and other APCs. IMO-2125 activates TLR-9 signals that ignite the immune response with the production of cytokines such as IFNγ, IL-6, and IL-12. The combination of this drug with anti-PD1 not only provoked a relevant reduction of the tumor in the primary site, but also showed an abscopal effect on distant sites as a result of the peculiar efficacy of IMO-2125 to prime the adaptive immune response.

A phase II clinical trial tested the efficacy of the combination of PD-1 inhibition (pembrolizumab) with a CXCR4 antagonist (BL-8040 also known as motixafortide) in patients with metastatic disease refractory to one or more previous lines of chemotherapy (186). BL-8040 is a small synthetic peptide that binds to and inhibits CXCR4 (187, 188). CXCR4 binds to its ligand CXCL12/SDF1, which is constitutively expressed in the bone marrow, and inhibits the mobilization of CXCR4 expressing immune progenitor cells. Indeed, numerous preclinical studies have shown that CXCR4 blockade through BL-8040 treatment stimulated mobilization of T, B, and NK cells from lymph nodes and bone marrow into the periphery (188, 189). Moreover, in murine models of lung cancer, BL-8040 also promoted selective reduction of Tregs (190).

BL-8040 monotherapy and in combination with pembrolizumab promoted an increase in the density of T cells (CD3^+^, CD4^+^, and CD8^+^) and activated cytotoxic T cells (CD8^+^ granzyme B^+^) and a decrease in immunosuppressive elements such as MDSCs in the TME.

This is in line with the results from a recent study demonstrating that the chemokine CXCL12 derived from CAFs impairs the trafficking of multiple immune cell types within the TME, thus favoring an immunosuppressive environment (191). The authors reported that BL-8040 in combination with pembrolizumab led to disease control in nearly a third of the patients with heavily pretreated pancreatic cancer (186). Successively, an expansion cohort of the study integrating BL-8040 and pembrolizumab with the NAPOLI-1 chemotherapy regimen was initiated (192). Enrolled patients had *de novo* metastatic PDAC and disease progression following first-line gemcitabine-based treatment. BL-8040 and pembrolizumab in combination with nanoliposomal irinotecan, fluorouracil, and folinic acid showed a potential for higher responses without added toxicity. Currently, the effects of BL-8040 and the anti-PD1 cemiplimab in combination with gemcitabine and nab-paclitaxel for the first-line treatment of metastatic PDAC have been tested in a phase II study (NCT04543071).

Another approach is targeting the CD40 member of the tumor necrosis factor family. CD40 is expressed on immune cells, and its stimulation through the use of agonists has been shown to increase anticancer activity (193, 194) by improving T-cell-dependent and -independent immune responses. Although preliminary, some encouraging data on the feasibility of the use of CD40 agonists are starting to be available. The phase I study NCT00711191 tested the therapeutic effect of the agonist CD40 monoclonal antibody (mAb) CP-870,893 in combination with gemcitabine in patients with advanced PDAC (195). Twenty-two patients with advanced chemotherapy-naïve PDAC, twenty of which with metastatic disease, were enrolled in the study. The results showed that combination of CP-870,893 with gemcitabine was well tolerated and provided some encouraging, although preliminary, evidence of efficacy. Following treatment, a systemic immune response was detected, characterized by leukocyte trafficking, cytokine production, and cellular activation. Moreover, thanks to metabolic imaging, the authors showed that many patients presented an overall decrease in the metabolic activity of the primary pancreatic lesion. Nevertheless, the responses of metastatic lesions to treatment were heterogeneous. These findings suggest that the CD40 agonist can potentially improve the efficacy of conventional therapies in PDAC treatment, but further studies are required (195).

Another phase I study (NCT03214250) tested the agonist CD40 mAb sotigalimab with gemcitabine and nab-paclitaxel, with or without anti-PD1 mAb nivolumab (196). The results of this study showed that this combination had clinical promise and a clinically manageable safety profile in patients with metastatic PDAC. Objective responses were documented in 14 of 24 dose-limiting toxicity-evaluable patients. Median PFS was 11.7 months (95% CI, 7.1 to 17.8), and median overall survival was 20.1 months (95% CI, 10.5 to not estimable). Moreover, systemic modulation of dendritic cells and B cells was detected, together with activation of CD4^+^ and CD8^+^ T cells. These data support the hypothesis that the addition of a CD40 agonist to chemotherapy activates innate and adaptive immune response in PDAC patients. This is also in line with observation from studies utilizing CD40 mAb in murine models of PDAC (197, 198). This approach is now being tested in a randomized phase II clinical trial (NCT03214250).

The modulating effect of CD40 agonists on TME is also confirmed by another clinical trial (NCT02588443). Specifically, the results showed decreased density of tumor stroma, increased DC activation, shift of TAM polarization from M2-like to M1-like, as well as increased T-cell infiltration, proliferation, and activation status (199).

Overall, these results suggest that CD40 agonists can potentially benefit patients by improving response to chemotherapy or immunotherapy and, since they act through distinct mechanisms compared to ICIs, may even provide an alternative for cancers refractory to ICIs.

**Figure 2:**
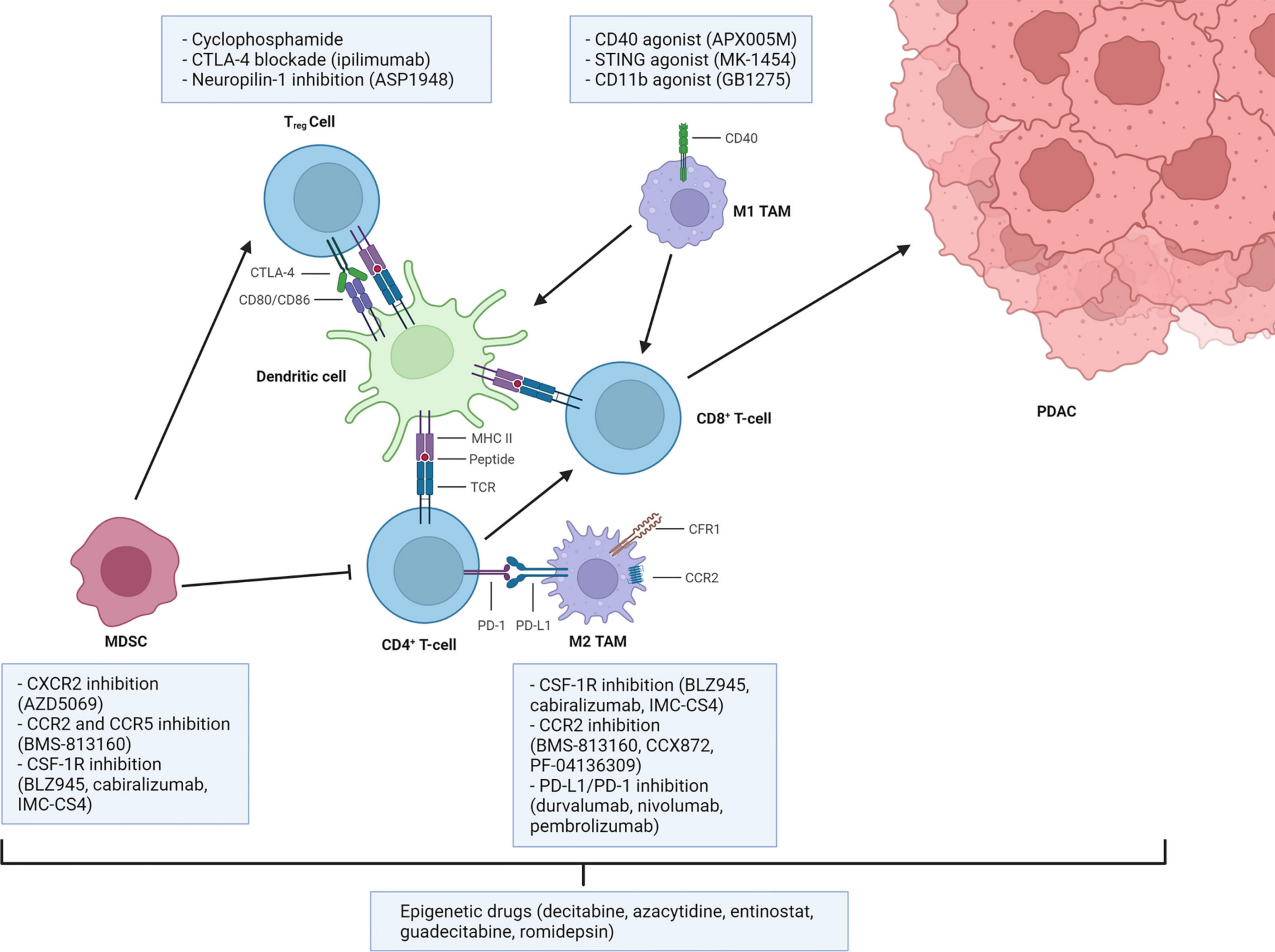
Main strategies to overcome myeloid and Treg-mediated immunosuppression. Dendritic cells or inflammatory macrophages (TAMs M1) sustain the antitumor immune response through antigen presentation. Myeloid-derived suppressor cells (MDSCs), anti-inflammatory tumor-associated macrophages (TAMs M2), and regulatory T (Treg) cells regulate these processes by exploiting inhibitory pathways, thus establishing an immunosuppressive tumor microenvironment. Some of the most clinically relevant therapeutic strategies available to target those pathways are reported. Created with BioRender.com.

Some of the possible strategies to overcome immunosuppression are presented in **Figure 2**.

The introduction of cellular immunotherapy has been a paradigm shift in cancer treatment.

Cell therapy based on chimeric antigen receptor (CAR) is one of the most studied approaches. CAR-T are genetically engineered T cells expressing specialized receptors that recognize and attack cancer cells (200), which are typically infused systemically. Although CAR-T therapy has shown promise in the treatment of hematological malignancies, its application in solid tumors has been hampered by a number of factors, such as immunosuppressive TME, sub-optimal survival, and ability of T cells to reach the tumor site, insufficient tumor infiltration, and limited choice of antigens (201, 202).

As previously described, the unique PDAC TME presents multiple challenges for the current therapeutic alternatives. CAR T cells are also affected by the numerous cellular components and extracellular matrix, which translates in a physical barrier impairing their detection and infiltration ability (203). Moreover, TME immune cells directly suppress T-cell activation through the release and the expression of a variety of factors that limit T-cell antitumor response (204). Another limit is represented by the deep heterogeneity observed in PDAC, both among the tumor cells as well as within the TME. This has drastically held up the identification of target antigens in PDAC (129). Despite these limitations, a number of targetable antigens suitable for cellular immunotherapy are currently being tested in both preclinical and clinical studies and include CEA, CD24, HER2, PSCA, MUC1, and MSLN (205, 206). Given the complexity of PDAC and its TME, and generally, to expand the use of CAR T cell therapy to solid cancers, cellular immunotherapies are also being explored in combination with other therapeutic approaches (207, 208). Recent studies have shown that chemotherapeutic drugs can be utilized in PDAC as priming agents before CAR T therapy in order to counteract the action of immunesuppressive cells, reduce autoimmunity, reduce tumor burden, sensitize cancer cells to immunotherapy, and improve CAR T cell survival rate *in vivo* (207).

In a phase I trial (NCT02159716), aimed at investigating the safety and efficacy of lentiviral-transduced CARs (209), subjects affected by solid cancers resistant to chemotherapy (PDAC, mesothelioma, and ovarian cancer) were administered anti-MSLN CAR T cells intravenously with and without cyclophosphamide pre-treatment. Indeed, the priming with chemotherapy was associated with an increase in CAR T cell expansion in peripheral blood, which peaked at day 14 after administration, but became undetectable after 6 months. Immune escape operated by tumors though upregulation of immune checkpoint receptors can also lead to CAR T cell inhibition (210).

The FDA has approved for solid tumors different checkpoint inhibitors, including mAbs against PD-1 and PD-L1 (211, 212). In the context of PDAC, CAR T cells against immune checkpoint inhibitors PD-1/PD-L1 were tested in PD-L1-overexpressing PDAC cells and in PDAC mouse models. Both CAR T cells induced tumor regression and reduced T-cell exhaustion (213). To overcome some of the limitations connected with the use of autologous CAR T cells, the implementation of allogeneic CAR T cells is being explored, as well as CAR NK cells and TIL therapy. Allogenic CAR T cells may offer a cheaper and more standardized alternative, which does not require individual-specific manufacturing. T cells can be collected from healthy donors, expanded, and stored, thus reducing time, cost, and variability for each treatment (214, 215). The main limitation with allogenic CAR T therapy is the potential risk for graft-versus-host disease (GvHD). Lack of compatibility between donor and recipient human leukocyte antigen (HLA) can lead to an immune response that will result in the elimination of the allogenic CAR T cells (216). Given its increased availability, next-generation sequencing is now being progressively more used to determine HLA compatibility; at the same time, gene editing technologies can be used to “hide” allogeneic CAR T cells from the host immune system by eliminating TCR expression (217).

CAR NK cells are also being evaluated as an alternative to allogenic CAR T cells (218). NK cells are components of the innate immune system that can recognize targets without prior sensitization, making them ideal candidates to deploy for therapeutic use against cancer (219). NK cells that recognize self-cells inhibit their own cytotoxic functions; therefore, more encouraging progresses have been made with allogeneic NK cell therapy in preclinical models and clinical trials. Indeed, it has been shown that autologous NK cells derived from cancer patients display less cytotoxicity compared to allogeneic NK cells, derived from healthy individuals (220, 221). In a recent study, Teng et al. employed, in a metastatic humanized pancreatic cancer mouse model, NK cells isolated from umbilical cord blood engineered to express a CAR construct recognizing prostate stem cell antigen (PSCA) and soluble IL-15 to improve antitumor response (222). The authors report an increase in cytotoxic function and survival, as well as reduced tumor growth and prolonged persistence of NK cells within the TME (up to 48 days).

Currently, two clinical trials (NCT02839954 and NCT03941457) are investigating the use of allogeneic NK cell infusions in PDAC, but no result has been published so far besides a case report from NCT03941457 showing that ROBO1-targeting NK cell infusions did not lead to serious toxicity (223). ROBO1 (Roundabout Guidance Receptor 1) mediates cellular responses to molecular guidance cues in cellular migration including neural axon guidance during development and has been found to be overexpressed in PDAC. While allogenic NK cells are a promising approach, one of the main limitations is the limited number of cells that a single donor can provide. Therefore, the use of NK cell lines is also being investigated (224). In a phase I clinical trial, CAR NK-92 cells directed against MUC1 and PD-1 were tested on a variety of cancers expressing both proteins (225). No severe adverse effects were observed, and out of 13 subjects, 9 presented stable disease, one presented progressive disease, while the other 3 were withdrawn from the study. In an orthotopic PDAC model, treatment with anti-ROBO1 CAR NK-92 cells was reported to synergize in combination with brachytherapy (226); moreover, CAR NK-92 cells were well tolerated when administered as a case study in an individual with metastatic pancreatic cancer, and the patient achieved stable disease for 5 months (223). In a recent study, Da et al. have investigated the antitumor efficacy of stimulator of interferon gene (STING) agonist cyclic GMP-AMP (cGAMP) in combination with CAR NK-92 cells targeting mesothelin in a preclinical mouse model of pancreatic cancer (227). The authors demonstrate that cGAMP can directly activate NK cells and enhance the sensitivity of pancreatic cancer cells to the cytotoxicity of NK cells. Moreover, the combination of cGAMP with CAR NK-92 cells targeting mesothelin improved antitumor efficacy (227).

Currently, three phase I/II clinical trials (NCT03941457, NCT03940820, and NCT03931720) are ongoing to evaluate the safety and efficacy of anti-ROBO1 CAR NK-92 cell therapy in PDAC and other solid tumors. In a more recent approach, CAR NK cells are manufactured from induced pluripotent stem cells (iPSCs). CAR iPSC NK cells are derived from triple-homozygous HLA donors, thus reducing the risk of rejection over multiple infusions, and with the advantage of working with a cell population that can grow indefinitely in an undifferentiated state *via* self-renewal (228, 229). Moreover, this approach also allows the increase of NK cell cytotoxicity through genetic engineering (230–236). Clinical and preclinical studies are still ongoing; however, CAR iPSC-NK cells could possibly provide a way for consistent production of NK cells with an identical phenotype.

TILs are a heterogeneous population of lymphocytes that naturally infiltrate solid tumors during the initial immune response (237). Briefly, TILs are isolated from a tumor biopsy and expanded *ex vivo*. The patient is then admitted to the hospital, to receive preconditioning non-myeloablative lymphodepletion, autologous TILs, and interleukin-2 (IL-2) infusion. Currently, the efficacy of TIL therapy in PDAC is being assessed in phase I and phase II clinical trials (NCT05098197, NCT03935893, and NCT03610490); however, TIL therapy has achieved positive clinical results in clinical trials for other cancers. The adverse effects reported are connected to the high dose of IL-2 required after infusion and to the lymphodepletion (238–241). Despite the limited clinical efficacy of cellular immunotherapy in PDAC, this field of research is still promising. Several strategies are being tested in order to overcome the challenges posed by the unique TME and heterogeneity of pancreatic cancer. Eventually, the development of off-the-shelf cellular immunotherapies will reduce manufacturing costs and time to treatment administration and result in overall less variability of the product.

Also, immunotherapy in combination with epigenetic therapy has recently been shown to be a promising approach (242, 243). Epigenetic alterations are prominent in PDAC (21) and may be involved in primary and acquired resistance to treatment by conferring fitness advantage to tumor cells (244). The first epigenetic drugs to be approved by the Food and Drug Administration (FDA) and European Medicines Agency (EMA) for certain hematological malignancies were inhibitors of histone demethyltransferases (DNMTis) and histone deacetylases (HDACis). However, first-generation epigenetic drugs like the DNMTis azacytidine and decitabine and the HDACis vorinostat and romidepsin have shown limited efficacy in the treatment of solid tumors (245). Second-generation compound drugs (the DNMTis zebularine and guadecitabine and the HDACis belinostat, panobinostat, tucidinostat, and valproic acid), while showing increased selectivity (245), have also shown considerable side effects. Recently, a new generation of epigenetic drugs is being developed and is entering clinical testing.

Epigenetic drugs have been tested in combination with other anticancer therapies, in order to overcome resistance and sensitize cancer cells to treatment.

In the context of immunotherapy of PDAC, it has been recently shown using the KPC mouse model that low-dose treatment with the hypomethylating drug decitabine (DAC) can potentiate the response to ICI therapy. The authors reported increased tumor necrosis, slowing of tumor growth, and increased numbers of CD4^+^, CD8^+^, PD-1^+^ TILs. However, the authors also reported a potentially unfavorable increase of M2 macrophages, following DAC treatment, that are predicted to antagonize ICI antitumor effects (243), thus suggesting that combination therapy using epigenetic drugs and immunotherapy can be further optimized.

In the future, new approaches will be developed involving a combination of next-generation epigenetic drugs and novel immunotherapy modalities, like vaccine-based and adoptive T-cell therapies (246, 247). The success of PDAC treatment will depend on the successful integration of genomic, epigenomic, and transcriptomic data in order to identify precise biomarkers for patient stratification and subsequent implementation of personalized strategies.

Precision medicine approaches have been nicely discussed in a very recent review. Hosein and colleagues have focused on current preclinical and clinical evidence to show promising combinatorial approaches, with the important conclusive message of future directions that could take into account side effects of PDAC treatments, with the aim to improve the quality of life for many patients (248).

### Keynote

PDAC is not completely strong and much vulnerability has been unveiled. Current research shows much more integrated approaches, to understand the disease from different points of view, but finally considering the unique context and unifying the huge efforts that many researchers are doing in the world. Last but not least, there is an urgent need for biomarkers to stratify patients and monitor therapies’ efficacy. Circulating cancer cells interact with immune cells influencing their function. The consideration of a systemic immune involvement should be a key point of view to understand surprising interactions.

## Conclusion

Despite the existence of a number of therapeutic options, PDAC remains among the diseases with the most urgent and prevalent medical need. The principal reason is the limited success of current treatments, which can be attributed to both late diagnosis and trouble in reaching and killing cancer cells. The challenging improvement of present therapeutic opportunities also harbors the necessity to identify targets for early diagnosis and novel drugs. To this aim, translational research focused in understanding the complicated connections among cells in the TME is more and more valuable to hypothesize novel treatment approaches. In closing, a strong prevention campaign for patients with high-risk factors and familiar predisposition for this cancer could be useful to avoid advanced disease.

## Author Contributions

AA, AO, CC, and IG wrote the first draft of the paper. AO prepared the figures. CC and IG supervised the work. All the authors contributed to revise and approve the final version submitted.

## Funding

The publication fee was funded by Biogem.

## Conflict of Interest

The authors declare that the research was conducted in the absence of any commercial or financial relationships that could be construed as a potential conflict of interest.

## Publisher’s Note

All claims expressed in this article are solely those of the authors and do not necessarily represent those of their affiliated organizations, or those of the publisher, the editors and the reviewers. Any product that may be evaluated in this article, or claim that may be made by its manufacturer, is not guaranteed or endorsed by the publisher.
